# What is the ‘spectral diet’ of humans?

**DOI:** 10.1016/j.cobeha.2019.06.006

**Published:** 2019-12

**Authors:** Forrest S Webler, Manuel Spitschan, Russell G Foster, Marilyne Andersen, Stuart N Peirson

**Affiliations:** 1 Laboratory of Integrated Performance In Design (LIPID), School of Architecture, Civil and Environmental Engineering (ENAC), École Polytechnique Fédérale de Lausanne (EPFL), Lausanne, Switzerland; 2 Department of Experimental Psychology, University of Oxford, United Kingdom; 3 Centre for Chronobiology, Psychiatric Hospital of the University of Basel, Switzerland; 4 Transfaculty Research Platform Molecular and Cognitive Neurosciences, University of Basel, Switzerland; 5 Sleep and Circadian Neuroscience Institute (SCNi), Nuffield Department of Clinical Neurosciences, University of Oxford, United Kingdom

## Abstract

Our visual perception of the world — seeing form and colour or navigating the environment — depends on the interaction of light and matter in the environment. Light also has a more fundamental role in regulating rhythms in physiology and behaviour, as well as in the acute secretion of hormones such as melatonin and changes in alertness, where light exposure at short-time, medium-time and long-time scales has different effects on these visual and non-visual functions. Yet patterns of light exposure in the real world are inherently messy: we move in and out of buildings and are therefore exposed to mixtures of artificial and natural light, and the physical makeup of our environment can also drastically alter the spectral composition and spatial distribution of the emitted light. In spatial vision, the examination of natural image statistics has proven to be an important driver in research. Here, we expand this concept to the spectral domain and develop the concept of the ‘spectral diet’ of humans.

**Current Opinion in Behavioral Sciences** 2019, **30**:80–86

This review comes from a themed issue on **Visual perception**

Edited by **Hannah E Smithson** and **John S Werner**

For a complete overview see the Issue and the Editorial

Available online 13th August 2019

**https://doi.org/10.1016/j.cobeha.2019.06.006**

2352-1546/© 2019 The Authors. Published by Elsevier Ltd. This is an open access article under the CC BY license (http://creativecommons.org/licenses/by/4.0/).

## What are the sources of light in the environment?

How we interact with light is mediated, altered, and challenged by physical filters from the macro-scale (atmosphere, vegetation, buildings, surfaces in the built environment) to the micro-scale (corneal surface, pupil reflex, and retinal structure). In the natural environment, the prevalent light is daylight, which is light from the sun filtered by the atmosphere, producing a broadband spectrum with characteristic ‘dips’ corresponding to absorption of light by molecules in the atmosphere. Variations in the atmosphere, as well as the Earth’s rotation, shape the spectral composition and the intensity of daylight [1,2]. Daylight availability differs between seasons, with longer days with higher-intensity light in the local summer and shorter days with lower-intensity light in the local winter, so that in addition to differences in vegetation, the outdoor environment undergoes seasonal changes in its spectral properties [3,4].

**Figure 1:**
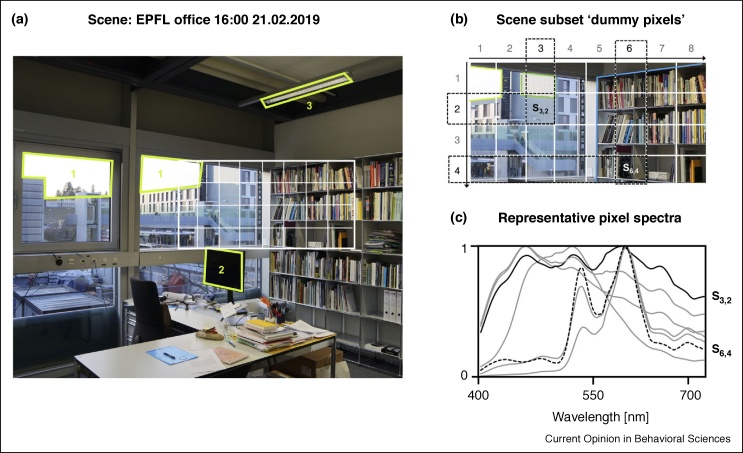
The modern light environment is complex, consisting of spectral data from a unique combination of illuminant spectra and reflected surfaces over space. In this office setting **(a)**, there is both direct and indirect (reflected) light. There are three direct sources: the sky (daylight), the computer screen, and the overhead fluorescent lighting. All other observed spectra are generated by source spectra interacting with objects in the scene, whose reflectance properties are not ideally diffuse (Lambertian). To simulate different representative spectral contributions in the scene **(b** and **c)**, we assumed reflectance values from the IES TM-30-15 Advanced Calculation Tool [5] and a dataset of 401 illuminant spectra [6]. The resulting spectrum over the pixel area comprises a weighted average of the incident spectra. As the pixel area approaches 1, each pixel corresponds to a unique spectrum in the scene.

Human innovation has produced dramatic changes in our light environment due to artificial lighting. Artificial light comes in many variants: incandescent, fluorescent, and most recently, light emitting diode (LED) lighting. These differ in the spectrum of the emitted light. Except in windowless rooms or rooms with full shading from outside illumination, indoor illumination always reflects a mixture of artificial and natural illumination as shown in Figure 1a.

Light emitted from any source also interacts with various types of matter before reaching an observer. Whether due to Rayleigh scattering in the atmosphere, or specular reflections from the glazed window panels of office buildings (see the example spectrum S_3,2_ in Figure 1c), light received at the eye is a product of interactions in our environment, which change the originally emitted source spectrum. Material properties alter the light environment in rather complex ways because surfaces in the real-world exhibit complex properties. We do not live in a Lambertian world, in which surfaces are matte. For this reason, estimating the effect of known illuminants on a human observer is non-trivial.

The visual world fortunately exhibits statistical regularities which naturally limit the set of unique combinations of illuminant spectra and reflective surfaces [7,8]. There are other regularities: the sky is in general brighter than the ground, and there is a bias towards dark contrasts in the sky but more balanced contrasts on the ground [9,10]. The same holds true of the spectra we are exposed to and a statistical approach can help to reduce the space of all possible spectra without needing to simulate complex scenes in a physically realistic way. The concept of standard illuminants (such as D65 ‘noon daylight’ or F11 fluorescent lighting) could be extended to whole spectral scenes, using a discrete basis set of spectral mixtures whose profiles would have been empirically derived and which we would be exposed to at a very high frequency in real life (e.g. see Figure 3b).

## What are the visual and non-visual effects of light on humans?

**Figure 2:**
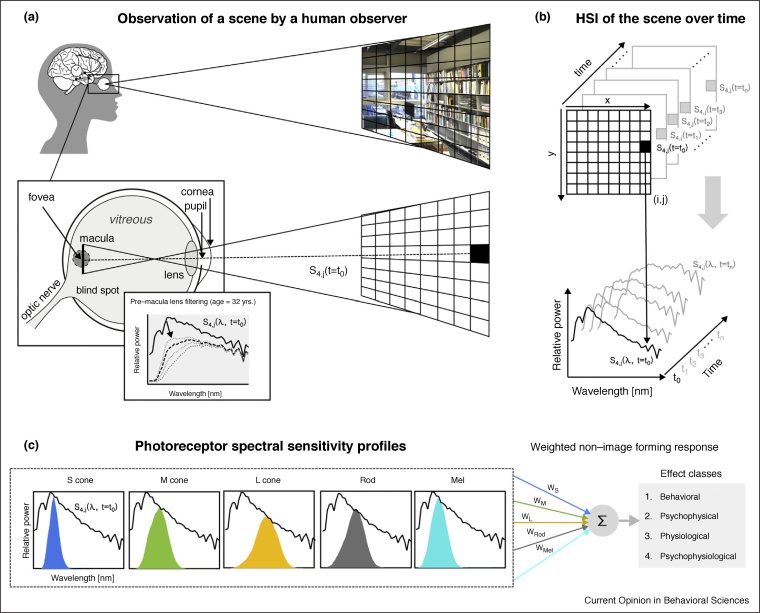
Overview of the human visual system’s response to a scene over the spectral range and over time. The human visual system is responsible for facilitating image forming and non-image forming processes. For a given ‘pixel’ in each scene, a spectrum enters the eye and is focused by the lens onto the retina **(a)** where different classes of photoreceptors are activated: each class of photoreceptors produces an output signal which is assigned a weight and ultimately leads to observable changes in the observer **(c)**. For that same ‘pixel’, a timeseries of hyperspectral images (HSIs) can be derived that represent the ‘spectral diet’ when projected onto the retina: at any instantaneous point in time t, a single HSI represents the visual stimulus over space spanned by the number of unique spectra coming from different direct and indirect sources **(b)**.

The human visual system comprises a variety of neural mechanisms which allows us to see space, detail, colour, and motion in the world, allows us to make value judgements about objects and actions, and to navigate from one place to another. In Figure 2a, we see how light information from a scene is projected onto the retina and subsequently processed to form an output signal leading to various physiological and psychophysical effects. Vision and visual perception are adaptive, responding to short-term, mid-term and long-term changes in the visual environment. Exposure to bright light can have long-lasting effects on visual perception. For example, exposure to one hour of sunlight can lead to shifts in colour matches, which can last for as long as five hours [11]. Exposure to filtered lights can also lead to long-lasting changes in colour perception [12]. In the laboratory, exposure to a background light can be used to selectively adapt and suppress specific photoreceptor mechanisms [13].

**Figure 3:**
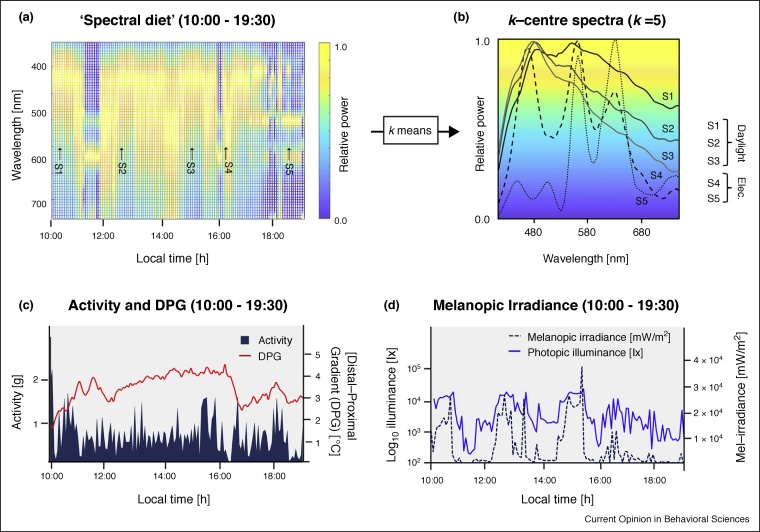
Visualising the spectral diet over a day. The spectral variation of light exposure over one work day (‘spectral diet’) is captured with an integrated wearable sensor. The experienced ‘spectral diet’ is visualised by plotting the normalised spectral information over time **(a)**, where variation in the relative power is shown with a gradient colour map. Accounting for statistical regularities, we conceptually propose to reduce the dimensionality of the spectral diet to a small group of representative spectra selected by running a *k*-means clustering algorithm on the set of all measured spectra **(b)**. This reduction shows that only a small set of spectra may effectively be able to describe the majority of the spectral information reached at the eye. The identification of common spectral ‘species’ might mean that we can use statistical regularities in the built environment to constrain the complexity of ‘decoding’ the spectral diet of humans. While biological parameters may reflect the relative effect of light exposure over time **(c)**, the illuminance conditions shown on a logarithmic scale highlight the variation in high intensity daylight and lower intensity electric lighting **(d)**. The device used to collect spectral data (a) includes a novel small-footprint array-based spectrometer developed by nanoLambda (Korea), which allows for variable sampling rates, data storage on-board or *via* bluetooth (approximate operating range: 5–40,000 l×).

By contrast, ‘non-visual’ or ‘non-image-forming’ effects are effects of light that are not related to vision or visual perception, such as circadian phase shifting [14], the acute suppression of melatonin [15], the regulation of pupil size [16] and the modulation of alertness [17^••^]. The term ‘non-visual’, however, is a misnomer, used as a catch-all term to describe a range of biological responses which relate to processes facilitated by the singular visual system, with all light-mediated signals originating from the retina. Similarly, the term ‘non-image-forming’ implies that there is no spatial specificity to these responses, even though from first principles, any photoreceptive system, no matter how broadly tuned it is in space, has a finite receptive field. There is also evidence suggesting that the recently discovered intrinsically photosensitive retinal ganglion cells (ipRGCs or pRGCs) modulate outputs from rods and cones and even play a role in pattern vision [18,19^••^]

Retinal photoreception proceeds from the absorption of photons by the photoreceptors, initiating a response in the form of an neutral pulse sent via the optic nerve to the suprachiasmatic nucleus (SCN) via a complex pathway where the circadian pacemaker is synchronised by the properties of the light stimulus [20]. The timing of circadian rhythms, regulated by the SCN, is then responsible for regulating sleep onset together with homeostatic processes. Destabilising the circadian pacemaker can result in the disruption of some or all of these processes, which in turn can result in changes in health, alertness, and wellbeing [21].

Retinal photoreceptors can be classified into three broad groups: rods, cones further classified as short (S), medium (M) or long (L) based upon their spectral sensitivity, and photosensitive retinal ganglion cells expressing the photopigment melanopsin. Rods and cones contribute directly to the image forming process and indirectly with pRGCs to communicate information about light/dark transitions and light levels [22,23^••^]. A complete understanding of the relative photoreceptor contributions (Figure 2c) and their combination (e.g. through opponency [24]) in the non-image-forming process is still lacking. Models fit to existing datasets [25] indicate that there may be a time-dependent relationship between the relative contributions of L and M cones and melanopsin when it comes to the sensitivity of melatonin suppression over time [26]. In addition, there is evidence in primates that S cones may inhibit pRGC excitation [27].

Ocular filters in front of the retina alter the spectrum of light reaching the retina [28], with the lens density being age-dependent [29] and the macular pigment being prominent only in the centre of the retina [30]. Pupil size varies depending on light level, relative photoreceptor activation (e.g. inhibition by S and M cones [31,32^••^]) and many other factors [33] between 2 and 8 mm in diameter (corresponding to a difference in retinal illuminance of only a factor of ∼16). As Barlow noted, it can “best be regarded as an inefficient homeostatic device if all it accomplishes is a reduction of input range from 10^11^ to 10^10^” [34]. But one order of magnitude change may make a difference in melatonin suppression [35], which exhibits a melatonin suppression [35], which exhibits a relatively sharp threshold [15]. This characteristic ‘step’ behavior is also reflected in recorded alertness data [36]. Retinal illuminance may also be modulated by squinting and the specific facial features, such as the brow, nose, and eyelid position, of an individual [37]. Finally, the retinal image is displaced frequently by head, eye and trunk movements [38, 39, 40].

## Why do we care about patterns of light in the environment?

Human exposure to light has changed profoundly. In the US in the 1800s, it is estimated that ∼90% of the population worked outside. By 2001, Americans spent 87% of their wake hours indoors [41]. The consequences of this substantial shift are poorly appreciated and the consequences on human health and performance remain unclear. Planners and designers of the built environment alone cannot predict how aspects of the built environment will impact life, but the light environment as a whole is fundamentally related to behavioural, psychophysical, and physiological responses. Light can be measured relatively easily, and new wearable sensors are making large-scale studies more feasible. At the city scale, access to light can be traced back to specific design interventions and strategies, e.g. in streetlighting. Understanding how features of the built environment facilitate light exposure is critical in understanding how modern living could be better aligned with human biology.

The exact impact of light on the totality of visual and non-visual responses is very difficult to predict. For example, a certain light exposure may directly affect physiology and behaviour by leading to an increase in alertness. That same light, however, may shift the circadian phase, which in turn *indirectly* could modulate alertness over a longer time scale, making an individual more prone to fatigue. Further, there might be an indirect effect of increased fatigue, causing a decrease in subjective wellbeing, all while conditions for visual comfort must be maintained. This ‘nesting’ of different neurophysiological processes and their entangled outcomes illustrates the complexity of separating indirect effects from direct effects. For this reason, it is a key challenge to develop methods of assessing the stability of the circadian system under different illumination patterns over extended periods of time (i.e. days and weeks).

Drawing an analogy to food, we refer to these patterns of light exposure as the ‘spectral diet’. The *absolute* spectral diet is the pattern of absolute spectral irradiance a human observer might receive over the course of a specific amount of time. The *relative* spectral diet refers to the spectral composition, or spectral quality, of the light received. Both absolute and relative spectral diets are important. The analogy to food diet here is the following: one might eat, over the course of the waking day, a fixed number of calories, for example, 2000 kcal. This number itself does not tell us how these 2000 kcal were spread across the different macronutrients. Similarly, an illuminance of 2000 lx received during daytime hours does not tell us how the different photoreceptors were affected by it. The concept of the spectral diet can be conceptualised and visualised by a continuum of hyperspectral images stacked together seen over time as illustrated in Figure 2b, which results in spectral data over the image span as visualised in Figure 3a.

We note that interrogating statistical regularities in the environment, and specifically in natural images, has a long history in neuroscience [42, 43, 44, 45]. The spectral diet is not simply a characterisation of the spectral environment for its own sake but could potentially also lead to inferences about the properties that an ‘ideal’ – or statistically optimal – observer’s photoreceptive system should have (e.g. spectral tuning, or temporal filtering properties).

## How can we measure patterns of light exposure in the environment?

Apart from being a useful concept to think about light input, how can we measure and characterise the spectral diet? Diurnal patterns of light exposure have been measured for healthy adults [46, 47, 48, 49, 50] and in psychiatric and neurological diseases and disorders (e.g. Seasonal Affective Disorder [51]; Alzheimer’s Disease [52]) using illuminance sensors. These sensors weigh the spectrum by the photopic luminosity function, which as a combination of L and M cones does not contain all information required to estimate the non-visual impact of a light.

A recent international standard (CIE S 026/E:2018) describes a retinally referenced framework for quantifying the effects of light from a given radiance or irradiance spectrum [53^••^]. This standard, based on previous proposals [54], allows for the determination of the extent to which the L, M and S cones, the rods, and melanopsin are activated by a given spectrum. For these calculations, the spectrum needs to be known or alternatively, sensors or imaging systems would need to incorporate the spectral sensitivities described by the standard. Another option is that spectral information could be computationally recovered (to some imperfect precision and accuracy) from a different set of sensors [55, 56, 57, 58], or different sensors could be used to recover information about cone, rod and melanopsin excitation [59] without a hyperspectral image.

What are the options of measuring spectral light exposure? Most spectrometers rely on a scanning linear CCD which limits their size and typically affordability. However, new technology using array-based sensors are now able to measure at 5 nm resolution within the visible range. These can be integrated into portable spectrometers worn at the face plane and may provide a more accurate assessment of the light reaching the eye (though with the caveats mentioned above, such as pupil size). These miniature spectrometers at the time of writing this review (mid 2019) on the market have footprints under 25 mm^2^. Compact circuit boards can be integrated with other sensors measuring activity, temperature, and GPS location to record near-continuous data with a long battery life. Some sample data collected with such a sensor are shown in Figure 3a.

The combination of new in-field calibration methods and weather sealing allow integrated spectral sensors to be used effectively under naturalistic, real-world conditions. The flexibility and relative low-cost of wearable sensors enable researchers to effectively measure light exposure on the neighbourhood and city scales and characterise the spectral diet in a large group of individuals. Data from such measuring campaigns could be then subjected to mathematical and computational models of the circadian system to determine circadian parameters such as amplitude and phase (e.g. [60^••^]), and be made available to other researchers using data sharing platforms (e.g. FigShare).

## Conclusion

The relationship between light and human biology is complex. Light intensity, duration, wavelength and timing, along with the individual history of light exposure and the age of the individual all need to be taken into consideration when characterising these relationships and an appropriate ‘spectral diet’. Since the beginning of human evolution, the circadian pacemaker has relied on daylight as the primary environmental zeitgeber. As the human environment changes to accommodate a 24/7 life style, our activity schedules are no longer tied to the availability of daylight. Artificial light, on the other hand, may manipulate timing and periodicity of our internal pacemaker. The statistical regularities in an individual’s spectral diet could be a first start in identifying how the spectral environment determines non-visual responses. But spectral data alone are not enough and for this reason we must also rely on ancillary data such as activity, core body temperature, sleep schedule, melatonin suppression, and others in order to contextualise incident light spectra and constrain models of visual and non-visual function.

## Funding

F.S.W. and M.A. are supported by the École Polytechnique Fédérale de Lausanne (EPFL). M.S. is supported by a Sir Henry Wellcome Trust Fellowship (Wellcome Trust204686/Z/16/Z) and a Junior Research Fellowship from Linacre College, University of Oxford. S.N.P and R.G.F are supported by the Wellcome Trust (098461/Z/12/Z and 106174/Z/14/Z) and the BBSRC (BB/I021086/1).

## Conflict of interest statement

M.S. has had the following commercial interests in the last two years (2017–18): Investigator-initiated research grants from f.lux Software LLC, and BIOS Lighting LLC; consulting contract with Seoul Semiconductors; speaker fees for invited seminars from Seoul Semiconductors and Apple. S.N.P. and R.G.F. have held consulting contracts with Dyson Ltd. R.G.F. is Director and Academic Founder of Circadian Therapeutics. F.S.W. and M.A. declare no conflicts of interest.

## References and recommended reading

Papers of particular interest, published within the period of review, have been highlighted as:

• of special interest
•• of outstanding interest

## References

[1] Judd D.B. (1964). Spectral distribution of typical daylight as a function of correlated color temperature. J Opt Soc Am.

[2] Spitschan M. (2016). Variation of outdoor illumination as a function of solar elevation and light pollution. Sci Rep.

[3] Jacobs N., Roman N., Pless R. (2007). Consistent temporal variations in many outdoor scenes. 2007 IEEE Conference on Computer Vision and Pattern Recognition.

[4] Webster M.A., Mizokami Y., Webster S.M. (2007). Seasonal variations in the color statistics of natural images. Network.

[5] Illuminating Engineering Society of North America (2015). IES Method for Evaluating Light Source Color Rendition (IES TM-30-15).

[6] Houser K.W. (2013). Review of measures for light-source color rendition and considerations for a two-measure system for characterizing color rendition. Opt Express.

[7] Maloney L.T. (1986). Evaluation of linear models of surface spectral reflectance with small numbers of parameters. J Opt Soc Am A.

[8] Vrhel M.J., Gershon R., Iwan L.S. (1994). Measurement and analysis of object reflectance spectra. Color Res Appl.

[9] Baden T. (2013). A tale of two retinal domains: near-optimal sampling of achromatic contrasts in natural scenes through asymmetric photoreceptor distribution. Neuron.

[10] Tkacik G. (2011). Natural images from the birthplace of the human eye. PLoS One.

[11] Jordan G., Mollon J.D. (1997). Adaptation of colour vision to sunlight. Nature.

[12] Neitz J. (2002). Color perception is mediated by a plastic neural mechanism that is adjustable in adults. Neuron.

[13] Stiles W.S. (1978). Mechanisms of Colour Vision.

[14] Dijk D.J., Lockley S.W. (2002). Integration of human sleep-wake regulation and circadian rhythmicity. J Appl Physiol (1985).

[15] Zeitzer J.M. (2000). Sensitivity of the human circadian pacemaker to nocturnal light: melatonin phase resetting and suppression. J Physiol.

[16] McDougal D.H., Gamlin P.D. (2015). Autonomic control of the eye. Compr Physiol.

[17••] Souman J.L. (2018). Acute alerting effects of light: a systematic literature review. Behav Brain Res.

[18] Ecker J.L. (2010). Melanopsin-expressing retinal ganglion-cell photoreceptors: cellular diversity and role in pattern vision. Neuron.

[19••] Allen A.E. (2017). Melanopsin contributions to the representation of images in the early visual system. Curr Biol.

[20] Hastings M.H., Maywood E.S., Brancaccio M. (2018). Generation of circadian rhythms in the suprachiasmatic nucleus. Nat Rev Neurosci.

[21] Birchler-Pedross A. (2009). Subjective well-being is modulated by circadian phase, sleep pressure, age, and gender. J Biol Rhythms.

[22] Lucas R.J. (2012). How rod, cone, and melanopsin photoreceptors come together to enlighten the mammalian circadian clock. Prog Brain Res.

[23••] Brown T.M. (2016). Using light to tell the time of day: sensory coding in the mammalian circadian visual network. J Exp Biol.

[24] Spitschan M., Lucas R.J., Brown T.M. (2017). Chromatic clocks: color opponency in non-image-forming visual function. Neurosci Biobehav Rev.

[25] Gooley J.J. (2010). Spectral responses of the human circadian system depend on the irradiance and duration of exposure to light. Sci Transl Med.

[26] Amundadottir M.L., Lockley S.W., Andersen M. (2016). Unified framework to evaluate non-visual spectral effectiveness of light for human health. Light Res Technol.

[27] Dacey D.M. (2005). Melanopsin-expressing ganglion cells in primate retina signal colour and irradiance and project to the LGN. Nature.

[28] Pokorny J., Smith V.C. (1997). How much light reaches the retina?. Colour Vision Deficiencies XIII.

[29] Pokorny J., Smith V.C., Lutze M. (1987). Aging of the human lens. Appl Opt.

[30] Snodderly D.M. (1984). The macular pigment. I. Absorbance spectra, localization, and discrimination from other yellow pigments in primate retinas. Invest Ophthalmol Vis Sci.

[31] Spitschan M. (2014). Opponent melanopsin and S-cone signals in the human pupillary light response. Proc Natl Acad Sci U S A.

[32••] Woelders T. (2018). Melanopsin- and L-cone-induced pupil constriction is inhibited by S- and M-cones in humans. Proc Natl Acad Sci U S A.

[33] Loewenfeld I.E., Lowenstein O. (1993). The Pupil: Anatomy, Physiology, and Clinical Applications.

[34] Barlow H.B. (1972). Dark and light adaptation: psychophysics. Visual Psychophysics.

[35] Gaddy J.R., Rollag M.D., Brainard G.C. (1993). Pupil size regulation of threshold of light-induced melatonin suppression. J Clin Endocrinol Metab.

[36] Cajochen C. (2000). Dose-response relationship for light intensity and ocular and electroencephalographic correlates of human alertness. Behav Brain Res.

[37] Sliney D.H. (1983). Eye protective techniques for bright light. Ophthalmology.

[38] Freedman E.G. (2008). Coordination of the eyes and head during visual orienting. Exp Brain Res.

[39] Land M.F. (2004). The coordination of rotations of the eyes, head and trunk in saccadic turns produced in natural situations. Exp Brain Res.

[40] Rucci M., Poletti M. (2015). Control and functions of fixational eye movements. Ann Rev Vis Sci.

[41] Klepeis N.E. (2001). The National Human Activity Pattern Survey (NHAPS): a resource for assessing exposure to environmental pollutants. J Expo Anal Environ Epidemiol.

[42] Barlow H. (2001). The exploitation of regularities in the environment by the brain. Behav Brain Sci.

[43] Simoncelli E.P., Olshausen B.A. (2001). Natural image statistics and neural representation. Annu Rev Neurosci.

[44] Rieke F., Rudd M.E. (2009). The challenges natural images pose for visual adaptation. Neuron.

[45] Geisler W.S. (2008). Visual perception and the statistical properties of natural scenes. Annu Rev Psychol.

[46] Cole R.J. (1995). Seasonal variation in human illumination exposure at two different latitudes. J Biol Rhythms.

[47] Hubert M., Dumont M., Paquet J. (2009). Seasonal and diurnal patterns of human illumination under natural conditions. Chronobiol Int.

[48] Okudaira N., Kripke D.F., Webster J.B. (1983). Naturalistic studies of human light exposure. Am J Physiol.

[49] Savides T.J. (1986). Natural light exposure of young adults. Physiol Behav.

[50] Scheuermaier K., Laffan A.M., Duffy J.F. (2010). Light exposure patterns in healthy older and young adults. J Biol Rhythms.

[51] Eastman C.I. (1990). Natural summer and winter sunlight exposure patterns in seasonal affective disorder. Physiol Behav.

[52] Campbell S.S. (1988). Exposure to light in healthy elderly subjects and Alzheimer’s patients. Physiol Behav.

[53••] CIE (2018). S 026/E:2018: System for Metrology of Optical Radiation for ipRGC-Influenced Responses to Light.

[54] Lucas R.J. (2014). Measuring and using light in the melanopsin age. Trends Neurosci.

[55] Chiao C.C. (2000). Characterization of natural illuminants in forests and the use of digital video data to reconstruct illuminant spectra. J Opt Soc Am A Opt Image Sci Vis.

[56] Connah D., Westland S., Thomson M.G.A. (2001). Recovering spectral information using digital camera systems. Color Technol.

[57] Hernandez-Andres J. (2004). Spectral-daylight recovery by use of only a few sensors. J Opt Soc Am A Opt Image Sci Vis.

[58] Valero E.M. (2007). Recovering spectral data from natural scenes with an RGB digital camera and colored filters. Color Res Appl.

[59] Cao D., Barrionuevo P.A. (2015). Estimating photoreceptor excitations from spectral outputs of a personal light exposure measurement device. Chronobiol Int.

[60••] Woelders T. (2017). Daily light exposure patterns reveal phase and period of the human circadian clock. J Biol Rhythms.

